# Difficulty in disengaging from threat and temperamental negative affectivity in early life: A longitudinal study of infants aged 12–36 months

**DOI:** 10.1186/1744-9081-8-40

**Published:** 2012-08-14

**Authors:** Atsuko Nakagawa, Masune Sukigara

**Affiliations:** 1 Graduate School of Humanities and Social Sciences, Nagoya City University, 1, Yamanohata, Mizuho-cho, Mizuho-ku, Nagoya, 467-8501, Japan

**Keywords:** Attention, Infant, Negative affect, Longitudinal study, Temperament

## Abstract

**Background:**

Attention disengagement is reportedly influenced by perceiving a fearful facial expression even in the first year of life. In the present study, we examined whether individual differences in disengaging from fearful expressions predict temperamental negative affectivity.

**Method:**

Twenty-six infants were studied longitudinally at 12, 18, 24, and 36 months, using an overlap paradigm and two temperament questionnaires: the Japanese versions of the revised Infant Behavior Questionnaire and Early Childhood Behavior Questionnaire.

**Results:**

The infants fixated significantly more frequently to fearful than to happy or neutral faces. The attentional bias to threat (i.e., the number of fixed responses on fearful faces divided by the total number of fixed responses on faces) at 12 months was significantly positively correlated with negative affect at 12 months, and its relations with negative affect measured later in development was in the expected positive direction at each age. In addition, a moderation analysis indicates that the orienting network and not the executive network marginally moderated the relation between early attentional bias and later fear.

**Conclusions:**

The results suggest that at 12 months, infants with more negative affectivity exhibit greater difficulty in disengaging their attention from fearful faces. We also found evidence that the association between parent-reported fear and disengagement might be modulated in the second year, perhaps because of the differences in temperamental control networks.

## Background

Visual-spatial attention systems can reportedly detect threat-related stimuli rapidly. The propensity to quickly detect the presence of threatening stimuli, such as snakes and angry faces, may be an important survival and adaptive mechanism. Threat-related stimuli (e.g., threat words or angry faces) may also cause a delay in disengagement [1], a tendency possibly increased by an individual’s elevated level of state anxiety. Further, using fearful facial expressions as stimuli, Georgiou et al. [2] showed that high trait-anxious people exhibited extended dwell time to threat-related stimuli. The inability to rapidly disengage from threat-related stimuli may keep cognitive resources focused on the stimuli and result in increased anxiety [1]. This trend might influence subsequent cognitive and emotional processing, which is likely to play an important role in shaping children’s cognitive representations of themselves, others, and the situation, from their earliest years [3].

Even in infancy, humans have been found to orient more quickly to threatening than to nonthreatening stimuli [4]. Recent studies have demonstrated that 7-month-olds disengaged their fixation significantly less frequently from fearful faces than from happy faces and control stimuli [5]. Moreover, Peltola et al. [6] found that the delayed withdrawal of attention reflected not a simple response to fearful wide-open eyes but rather an enhanced sensitivity to facial signals of threat. Fearful expressions also caused greater heart rate deceleration responses in 7-month-old infants during the first 1000 ms of face viewing [7]. Leppänen et al. [7] concluded that emotion–attention interactions such as those displayed by adults can also be observed early in life.

Growing evidence suggests that attentional bias to threat plays a causal role in individual differences in emotional vulnerability [8,9]. Lonigan et al. [10] considered temperament’s contributions to childhood disorders. They extracted factors similar to those drawn previously from childhood self-reported items that assess emotionality and attention and described automatic attention allocation mechanisms linked to negative affectivity, which may have an effect on both the daily experiences of children and their proneness to future negative experiences. Thus, the association between negative affectivity and anxiety pathology could be mediated by the attentional bias to threat.

Going beyond the individual mechanism of anxiety-related information processing, Fox et al. [11] proposed a model of plasticity for affective neurocircuitry, describing how genetic disposition and environmental circumstances may interact. Thus, a child’s fearful temperament elicits and is elicited by the caregiver’s insensitivity and intrusiveness to shape the attentional bias to threat and the neural systems involved in this bias (i.e., ventral prefrontal cortex-amygdala circuitry). Fox et al. [11] further suggested that exaggerated attentional bias to threat may cause the emergence and maintenance of anxious behaviors.

On the basis of a meta-analysis of 172 studies, Bar-Haim et al. [12] pointed out that although attentional bias to threat may largely contribute to the development and maintenance of anxiety over time, the possibility of a causal link between the two has been insufficiently investigated. Recently, longitudinal studies of very young children have examined the relation between attentional bias to threat and later socioemotional outcome or risk of psychopathology [13,14]. Results indicate the moderating roles that attention played in anxiety development. However, the youngest participants in these studies were 24 months old [13], and the dot-probe task used for very young children did not necessarily measure the ability to disengage [14]. Therefore, further research is needed to fill these gaps in the previous studies in order to understand the initial structure and function of anxiety-related information processing.

For the reasons stated above, we conducted a longitudinal study of infants approximately 12–36 months old. Fear develops by the end of the first year of life, and fearful infants show inhibition of motor approach [15]; hence, we studied infants from the end of their first year. The purpose of this study was, first, to confirm the infants’ greater difficulty in disengaging attention from fearful faces than from happy or neutral faces [5]. In the overlap task that we used following Peltola et al. [5], infants were required to disengage their fixation from a centrally presented facial expression and shift attention to a peripheral target.

Second, we examined the relationship between individual differences in fear or negative affectivity and the attentional bias to threat in early life. For this purpose, we examined individual differences in fear or negative affectivity through a revised Infant Behavior Questionnaire (IBQ-R Japanese version [16]) administered at 12 months and the Early Childhood Behavior Questionnaire (ECBQ Japanese version [17]) administered at ages 18–36 months. Since an increased number of fixation responses (i.e., no movement) with a centrally presented fearful face has been given as evidence of the effect of fearful faces on attentional disengagement [5], we computed an index of the attentional bias to threat-related stimuli on the basis of these fixation responses. Negative affectivity (i.e., the reactive component of temperament) is considered relatively easy to change early in life. Effortful control reflects a voluntary component of attention and undergoes significant development in the second year of life and later [18]. In the first year of life, the association between a high level of negative affectivity and an attentional bias to threat would involve a mainly reactive temperamental component and would be easier to notice. In the second and third years, however, this connection might be modified by control systems such as effortful control. Children and adolescents, who are considered high in negative affectivity and low in effortful components of temperament, are reported to demonstrate a significant attentional bias in favor of threat stimuli [19].

Third, we conducted a moderation analysis to examine if an effortful control moderates the link between attentional bias at 12 months and temperament at 36 months. In infancy, a brain network involved in orienting to sensory events may provide the chief means of self-regulation. This orienting network involves areas of the inferior and superior parietal lobes and the frontal eye fields. Later in childhood, however, the executive attention system, including the anterior cingulate, insula, and areas of the basal ganglia, becomes dominant as a mechanism of self-regulation [20]. Effortful control as a temperamental construct is considered to reflect the functioning of a neutrally based executive attention. The IBQ-R orienting score measures an orienting attentional network, and the ECBQ effortful score assesses an executive attention network. Therefore, we included both the orienting score of the IBQ-R (at 12 months) and the effortful control score of ECBQ (at 24 months). The interactive (moderating) effect was tested by using centered variables in hierarchical regression.

We hypothesized that effortful control buffers the link between attentional bias and the variables of fear or negative affect. Specifically, we predicted that the correlation between the 12-month attentional bias score and 36-month temperament score (fear or negative affectivity) would be stronger among toddlers low in effortful control. On the other hand, toddlers with high orienting scores might interact in the opposite manner. Because high scores on the latter are partially due to a tendency to focus for long periods, toddlers who attend to threat might be particularly likely to continue focusing on threatening stimuli, thus possibly increasing the likelihood that they will be fearful at 36 months.

## Method

### Participants

Twenty-six infants (15 boys, 11 girls) with no history of perinatal or postnatal difficulties were recruited through local maternity groups in Nagoya, Japan’s third largest metropolitan area, located near the center of the country. Criteria for participation in the study were no known complications of birth or other causes, having been carried to full term (more than 37 weeks gestation), and normal birth weight (2500 g–4000 g). The infants were longitudinally assessed four times (at 12, 18, 24, and 36 months of age) through eye movement recordings and behavior questionnaires filled out by their caregivers. All caregivers were Japanese mothers who gave informed consent on behalf of their infants before the experiments. The study was approved by the Ethics Committee of Nagoya City University (No. 07007) and accorded with the ethical standards specified in the 1964 Declaration of Helsinki. One participant at 18 months, four at 24 months, and one at 36 months of age could not participate in the experiments for personal reasons (e.g., birth of a sibling or father’s work reassignment).

### Procedure

#### Eye movement recording

Each participant sat in a baby chair in a semidark area, 65 cm from the color monitor of an AV tachistoscope (IS-702). During the eye movement recording, the participant’s mother was nearby but stayed out of sight. An experimenter outside the semidark area monitored the participant’s eye movements through a low-angle CCD near-infrared video camera (ELMO CN43H) positioned in front of the participant and controlled the stimulus presentation by means of a microcomputer (FMV-S167). The stimuli presented were superimposed synchronously on video images of the infant’s eye movements by a digital image processor (FOR-A, MF-310) and were then recorded on videotape (SONY DSR-11), which was subsequently used for off-line video coding.

#### Facial expression overlap task

At the beginning of each trial, the experimenter displayed a central fixation attractor on the monitor in front of each infant or toddler by pressing a key. While the infant looked at the attractor, the experimenter presses another key, causing the facial expression stimulus (i.e., a happy, fearful, or neutral face) to replace the fixation attractor in the center. The central stimulus remained visible throughout the trial. Subsequently, (200 ms afterward), a peripheral stimulus was presented for 2600 ms at approximately 30 degrees to either the left or right of the central fixation point. The experiment comprised a total of 24 trials, with 12 left and 12 right targets presented in a pseudorandom order.

#### Stimuli

The central fixation attractors and peripheral targets comprised brightly colored abstract figures, which were animated and subtended at a visual angle of 5 degrees. Each central fixation attractor was accompanied by a sound. The central facial stimuli used were from *Japanese and Caucasian Facial Expressions of Emotion*[21]. For each expression (i.e., happy, fearful, or neutral), color images of two male and two female models were displayed on the monitor, and the peripheral stimuli were reflected in a first-surface mirror on the left or right side at approximately 30 degrees from the central fixation point. The facial stimuli appeared at a vertical and horizontal visual angle of 9 and 6 degrees, respectively.

#### IBQ-R and ECBQ

The following instruments were used to assess the frequency of occurrence, over the previous one or two weeks, of temperament-related behaviors on a 7-point Likert scale ranging from *never* to *always*. The IBQ-R was administered at 12 months, and the ECBQ at 18, 24, and 36 months. The IBQ-R included 191 items yielding 14 scales, while the ECBQ consisted of 201 items containing a total of 18 scales. Factor analyses of the IBQ-R and ECBQ scale scores identified a three-factor solution: Positive Emotionality/Surgency, Negative Affectivity, and Regulatory Capacity/Orienting [22]. The following αs are based on Nakagawa and Sukigara [16] and Sukigara et al. [17]. In the IBQ-R, scales of Distress to Limitations (*α* = .82), Sadness (*α* = .83), Falling Reactivity (negatively; *α* = .80), and Fear (*α* = .92) primarily loaded on Negative Affect. The Orienting factor of IBQ-R was primarily contributed by Attention/Duration of Orienting (*α* = .74), Low Intensity Pleasure (*α* = .78), Soothability (*α* = .61), and Cuddliness (*α* = .77). In the ECBQ, scales of Frustration (*α* = .80), Sadness (*α* = .78), Discomfort (*α* = .65), Motor Activation (*α* = .69), Fear (*α* = .72), Soothability (negatively; *α* = .87), Shyness (*α* = .82), and Perceptual Sensitivity (*α* = .81) primarily loaded on Negative Affect. Moreover, the effortful control factor of ECBQ was primarily defined by Attention Focusing (*α* = .89), Inhibitory Control (*α* = .85), Attention Shifting (*α* = .66), Low Intensity Pleasure (*α* = .71), and Cuddliness (*α* = .74).

At the end of each experimental session (at 12, 18, 24, and 36 months of age), the mothers were given a temperament questionnaire (IBQ-R or ECBQ) to complete at home and mail back.

#### Data analysis for video coding

Videotapes of the eye movements were coded off-line by two independent coders not directly involved in the experiment. The number of scorable trials was calculated for each participant of each age. On average, the following numbers of trials were excluded from the 24 trials owing to the infant’s failure to fixate on the central facial stimuli before the target presentation: 2.95 ± 2.68 for 12-month-olds, 2.20 ± 2.75 for 18-month-olds, 2.26 ± 2.32 for 24-month-olds, and 1.43 ± 1.75 for 36-month-olds. Most of these cases involved failure to fixate on the first central attractor presented for 7 sec.

Of the scorable trials, those in which the infant gaze followed the direct path from the central face to the peripheral target were coded as responses toward targets. If an infant did not move his/her eyes from the central face during the trial, the response was coded as a fixation response on the face. There were also failures to respond to targets (e.g., infants looked to the opposite side of the target or made eye movements with latencies < 200 ms after the target onset). Probabilities of both the responses toward the targets and fixation responses were calculated by dividing the number of times the response occurred by the number of scorable trials.

The reaction time (RT: in video frames, 33 ms per frame) was recorded by carefully choosing the first frame in which an eye movement to a target was detected. We defined response latency as the elapsed time between the presentation of the peripheral target and the beginning of the movement of the infant’s gaze toward the peripheral target. Head movements were not coded in this study.

An examination of the inter-rater reliability between the two coders showed .96 agreement regarding their classification of responses (i.e., disengagement responses to targets, responses to stay fixated on the face, and failures to respond to targets) and .94 correlation between the RTs of the disengagement responses. Because participants with less than two scorable responses (responses to targets or fixation responses) under any of the experimental conditions were excluded from the analysis, three infants at 12 months, one at 18 months, three at 24 months, and one at 36 months were deemed ineligible as they showed excessive fussiness or crying.

## Results

To treat the uneven distribution of the dependent variables, we applied a log linear transformation to latencies and an arcsine transformation to response probabilities. In addition, a number of correlations were determined in this study; hence, to control for Type 1 error, a *p*-value of less than .01 was considered to indicate a statistically significant correlation.

Table 1 presents the means and standard deviations of both the probabilities of responses to targets and those of fixation responses on the face in each experimental condition across age. Since the probabilities of responses to targets (disengagement) were relatively low and they varied across conditions, the latency data, which are based on a low number of trials per cell and not comparable across conditions, are of limited use for a longitudinal analysis. Because the probability of fixation responses on the face (no movement) and that of responses to the target are inherently inversely related, we used fixations as a metric of infants’ “failure to disengage.” This also excludes failures to respond to targets mentioned above.

**Table 1:** Mean probabilities of responses to targets and fixation responses (Following Arcsine Transformation)

		** *Age* **			
	** *Expression* **	** *12 months* **	** *18 months* **	** *24 months* **	** *36 months* **
Probability of responses to peripheral target	Fearful	.410 (.392)	.431 (.286)	.513 (.373)	.683 (.408)
	Happy	.513 (.372)	.507 (.241)	.469 (.245)	.733 (.450)
	Neutral	.621 (.390)	.600 (.258)	.705 (.382)	.745 (.368)
Probability of remaining fixed Responses	Fearful	.522 (.376)	.514 (.261)	.546 (.316)	.295 (.313)
	Happy	.435 (.282)	.395 (.156)	.455 (.285)	.234 (.181)
	Neutral	.352 (.285)	.328 (.148)	.334 (.311)	.260 (.224)

Note. Standard deviations are given in parentheses.

### Probabilities of fixation responses on the face

To confirm that infants have greater difficulty in disengaging attention from fearful faces than from happy or neutral faces, we conducted a 3 (facial expression: fearful, happy, neutral) × 4 (age: 12, 18, 24, and 36 months) within-subject analysis of variance (ANOVA; Table 1) on the probability of fixation responses on the face. The main effects of facial expression and age were significant (*F*(2, 30) = 12.91, *p* < .001; *F*(3, 45) = 2.98, *p* < .05, respectively). Infants fixated with greater probability on fearful (*M* = .472) than on neutral (*M* = .319; *p* < .001) or happy faces (*M* = .382; *p* < .05). Further, no significant difference was found between neutral and happy conditions. Regarding the main effect of age, a significant difference existed only between 24 months (*M* = .447) and 36 months (*M* = .263; *p* < .05).

### Intercorrelations across ages

Table 2 presents the stability of individual variations in attention disengagement (the probabilities of fixation responses) from 12 to 36 months. We found significant correlations between 18 and 24 months and between 24 months and 36 months in the fearful condition. Table 3 shows the correlations between temperamental scores (Fear, Negative Affectivity) from 12 to 36 months. Regarding Negative Affectivity, all correlations between each age and the next proved significant and there was also a correlation between 18 and 36 months. Our results demonstrated some stability in the temperamental scores over these ages.

**Table 2:** Intercorrelations between probabilities of fixed responses across time

		** *18 months* **	** *24 months* **	** *36 months* **
Fearful	12 months	−.180	−.009	−.112
	18 months	-	.587^**^	.324
	24 months	-	-	.577^**^
Happy	12 months	.137	.112	.106
	18 months	-	.344	−.007
	24 months	-	-	.113
Neutral	12 months	−.132	−.036	−.254
	18 months	-	.057	−.051
	24 months	-	-	.492

^**^p < .01.

**Table 3:** Intercorrelations between temperamental scores across time

		** *18 months* **	** *24 months* **	** *36 months* **
Fear scale	12 months	.477	.020	.359
	18 months	-	.292	.338
	24 months	-	-	.218
Average score of scales loading on Negative Affect	12 months	.584^**^	.370	.344
	18 months	-	.670^**^	.578^**^
	24 months	-	-	.735^**^

^**^p < .01.

### Relation between attentional bias and temperament

To examine the relation between individual differences in the attentional bias to fearful faces and fearfulness or negative affectivity, correlation coefficients were calculated. The relevant index for attentional bias to threat could be response variables to fearful expression, controlling for the overall level of the infants’ failure to disengage from a face regardless of its expression. Therefore, we calculate the following proportion score as an index of attentional bias to threat: the number of remaining fixed responses on fearful faces divided by the total number of remaining fixed responses on faces. Average indices were .363 (*SD* = .166) for 12 months, .434 (*SD* = .254) for 18 months, .469 (*SD* = .312) for 24 months, and .238 (*SD* = .199) for 36 months. Table 4 presents the correlation coefficients between the index of attentional bias to threat and the temperamental fear or negative affectivity scores on the IBQ-R or ECBQ [22]. As a result, the relationship between the present attentional bias index toward threat and the degree of negative emotions was significant at 12 months (.527). The 95% confidence interval for this is .144 to .770.

**Table 4:** Correlation coefficients between temperamental scores and attentional bias index to fearful expression

		** *Attentional bias index to fearful expression* **			
		** *12 months* **	** *18 months* **	** *24 months* **	** *36 months* **
Fear scale (IBQ-R or ECBQ)	12 months	−.020	-	-	-
	18 months	.240	−.231	-	-
	24 months	−.100	−.335	−.262	-
	36 months	.174	−.463	.105	.017
Average score of scales loading on Negative Affect	12 months	.527**	-	-	-
	18 months	.403	−.083	-	-
	24 months	.222	−.286	−.023	-
	36 months	.309	−.276	−.125	.146

^**^p < .01.

Results indicate that 12-month-old infants with more negative affectivity show more difficulty in disengaging attention from fearful faces. Attentional bias to threat at 12 months was related not to parent-reported fear but to a broad factor of negative affectivity.

As the response probability was relatively high at 36 months (Table 1), we applied latency in the fearful expression condition as an attentional bias index at 36 months (*M* = 1150.85 ms, *SD* = 47.04). While no relationship was observed with negative affect, we found that latency in the fearful expression condition was positively correlated with the scores on the Fear scale (*r* = .516, *p* = .012).

### Moderating role of temperamental control

We examined whether temperamental control moderates the relation between fearful attentional bias at 12 months and temperament at 3 years. In hierarchical multiple regression analyses, attentional bias to threat (the number of fixed responses on fearful faces divided by the total number of fixed responses on faces) at 12 months, orienting score at 12 months, and effortful control score at 24 months were investigated as predictors of the temperament at 36 months. Independent variables were centered at their means prior to the analysis. Table 5 summarizes these results.

**Table 5:** Moderation Analysis: Temperamental control variables as moderators in the relationship between attentional bias to threat at 12 months and temperamental score of fear and negative affects at 36 months

**Predictor**	**Fear at 36 months**		
	**b(SE)**	**β**	**t**
Step 1 (ΔR^2^ = .309, R^2^ = .309, F(3,19) = 2.38)			
Attentional Bias to threat at 12 M	1.05 (1.03)	.21	1.01
Orienting at 12 M	−.26 (.39)	−.15	−.67
Effortful control at 24 M	−.77 (.37)	−.45	−2.03^†^
Step 2 (ΔR^2^ = .140 R^2^ = .449, F(5,19) = 2.28)			
Attentional Bias to threat at 12 M	2.07 (1.35)	.41	1.52
Orienting at 12 M	−.15 (.38)	−.08	−.39
Effortful control at 24 M	−.95 (.40)	−.56	−2.37^*^
Attentional Bias to threat at 12 M × Orienting/Regulation at 12 M	5.45 (3.02)	.44	1.80^†^
Attentional Bias to threat at 12 M × Effortful control at 24 M	2.18 (5.19)	−.11	.42
**Predictor**	**Negative affects at 36 months**		
	**b(SE)**	**β**	**t**
Step 1 (ΔR^2^ = .213, R^2^ = .213, F(3,19) = 1.44)			
Attentional Bias to threat at 12 M	1.03 (.69)	.33	1.48
Orienting at 12 M	−.12 (.26)	−.11	−.47
Effortful control at 24 M	−.30 (.25)	−.28	−1.19
Step 2 (ΔR^2^_=_ .0, R^2^ = .213, F(5,19) = .76)			
Attentional Bias to threat at 12 M	1.04 (1.01)	.33	1.02
Orienting at 12 M	−.12 (.28)	−.11	−.43
Effortful control at 24 M	−.30 (.30)	−.28	−1.01
Attentional Bias to threat at 12 M × Orienting/Regulation at 12 M	.06 (2.26)	.00	.02
Attentional Bias to threat at 12 M × Effortful control at 24 M	.03 (3.89)	.00	.00

^*^p < .05; ^†^*p* < .10.

**Figure 1:**
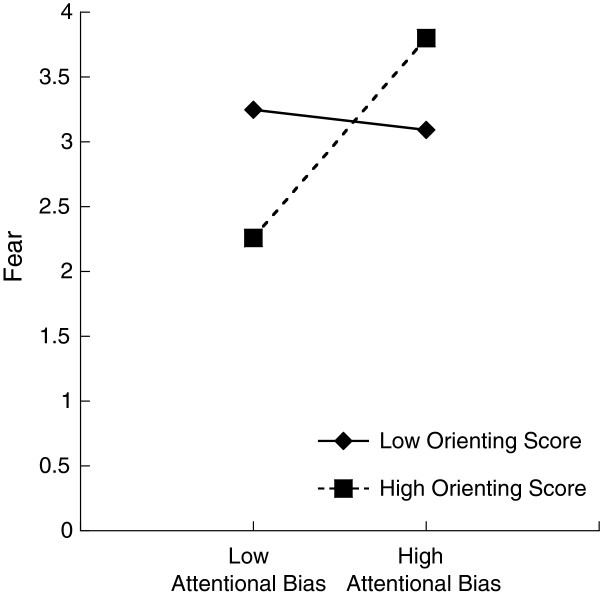
Joint effect of attentional bias to threat and temperamental orienting score on 36-month fear.

As can be seen in Table 5, in a main effects model of fear, effortful control only showed a trend toward relating to fearful temperament (*b* = −.77, *t* = −2.03, *p* = .059). Although the addition of interaction terms resulted in nonsignificant change in the model (Δ*R*^2^ = .140, *p* = .20), only the attentional bias to threat × orienting interaction term was marginal (*b* = 5.45, *t* = 1.80, *p* = .093). Thus, toddlers who showed higher effortful control were less fearful, but effortful control at 24 months did not moderate how attentional bias to threat at 12 months related to fear at 36 months. Yet, as depicted in Figure 1, the trend of the interaction effect indicates that the attentional bias to threat at 12 months predicted fear at 36 months with a higher level of orienting score. With a lower level of orienting score, the attentional bias to threat at 12 months did not predict fear at 36 months. This trend is consistent with our expectation. Models for negative affectivity are nonsignificant, and neither the main effect nor the interaction term is significant.

## Discussion

In this study, we investigated relations among disengagement from threatening stimuli, negative affectivity, and effortful control in infants across the second and third years of life. The infants demonstrated greater difficulty in disengaging their attention from fearful faces than from happy or neutral faces. The ability to disengage from fearful faces at 12 months was significantly related to negative affect only at 12 months, although it was positively related with negative affect across the age range of 12–36 months (Table 4). In addition, our results marginally indicate that it was not the executive network but rather the orienting network that moderated the association between early attentional bias and later fear.

These results indicate that infants with higher negative emotionality exhibit more extended dwell time for fearful facial expressions at 12 months. Individuals with strong attentional bias to threat might experience more stimuli as aversive, or their negative reactivity might be triggered by more stimuli [10]. In that case, earlier intervention—namely, the caregiver’s efforts in soothing at an earlier stage—might be necessary for infants high in negative affectivity. On the other hand, at 18, 24, and 36 months, no significant positive correlation was observed between temperament and the probability of the fixated responses to threat (Table 4). These results might be due to the development of attentional control over a relatively reactive component of temperament.

The subjects’ probability of responding to peripheral stimuli was higher at 36 months than earlier (Table 1), which is consistent with the results of ANOVA for probabilities of fixation responses on the face. Therefore, we applied RT as an index and found a positive correlation between the Fear scale and RT in the fearful condition. Rothbart et al. [23] mentioned that younger infants present relatively undifferentiated distress, but later it is possible to differentiate anger/frustration from fear. Thus, it is understandable that at an early stage, attentional bias is related not to fear but to overall negative affectivity. These data also suggest that if we apply adequate individual indices for each age, the relation between temperament and attentional bias to threat is found across the age range of 12–36 months. In any case, just as Kiel and Buss [13] found attention toward threat in the toddler years to be a predictor of social inhibition in kindergarten, the present study revealed a predictive role of attentional bias to threat at 12 months.

Temperamental control systems may play an important role in moderating the link between attentional bias to threat and later fear or negative affectivity. Posner et al. [20] argued that during infancy, control is principally carried out by the orienting brain network, whereas by three to four years of age this control shifts primarily to the executive network. With regard to effortful control, we only found a main effect relating to fear at 36 months. A negative relation between effortful control and negative affect has been reported consistently [23]. The orienting attention network marginally moderated the association between early attentional bias to threat and later fearful temperament (Figure 1). As expected, attentional bias at 12 months seems to predict fear at 36 months for children who had high orienting scores on the IBQ-R but not for those with low scores. Since high orienting scores are partially attributed to a tendency to focus for a long period (measured by the subscale of the duration of orienting), toddlers who attend to threat might be particularly likely to continue interacting with threatening stimuli, thus possibly increasing the likelihood that they will be fearful at 36 months. One possible reason that we found no interaction of effortful control is that at 36 months, both orienting and executive networks perform regulatory functions, while the executive network was subdominant earlier in life.

The current results were consistent with those of Peltola et al. [5,6] and Leppänen et al. [7], indicating that, as in adults, attention disengagement was influenced by fearful faces in 7-month-old infants (e.g., average disengagement latencies for fearful face, *M* = 674 ms; happy face, *M* = 554 ms; neutral face, *M* = 540 ms; [6]). These studies showed the face alone for 1000 ms at the center before presenting the peripheral stimulus, which was approximately 14 degrees to either the left or right of the center. On the other hand, the current study presented the face alone for 200 ms in the center and the peripheral stimuli at approximately 30 degrees from the center. Two hundred milliseconds is a psychophysical threshold of face visibility during infancy [24]. These differences in experimental conditions may have influenced RTs in the experiment as it took longer for the participants to disengage attention from the central faces (at 36 months, fearful face, *M* = 1150 ms; happy face, *M* = 1023 ms; neutral face, *M* = 930 ms).

The present results should be considered in light of several limitations. First, because many of the infants participated in very few trials, our data may not adequately represent a child’s attentional bias or difficulty to disengage from a threat. Second, to control for Type I error regarding correlation coefficients, we decide to treat a *p*-value less than .01 as statistically significant. However, this is less conservative than applying the Bonferroni correction. Further, our sample size was rather small.

## Conclusion

In summary, the longitudinal study of infants in the age range of 12–36 months revealed that the attentional bias to threat is preserved over time in infancy and childhood. The results also indicated that individual differences in the strength of that bias depend upon parent-reported temperamental negative affectivity or fearfulness in early life. Moreover, the attentional bias to threat may possibly interact with the temperamental orienting network. Future research should examine whether individual differences in attentional bias to threat emerging early in infancy predict later emotional traits. Investigation of the period before the effortful control becomes dominant might provide valuable information for parenting, early education, and child psychiatry.

## Competing interests

The authors declare that they have no competing interests.

## Authors’ contributions

Both authors conceived of the study and conducted the experiments. MS administered the instruments and analyzed data. AN mainly collected data and prepared the initial draft of the manuscript. Both authors approved the final manuscript.
